# Changes in mean corpuscular volume after erythropoiesis-stimulating agent treatment are associated with renal outcomes in non-dialysis-dependent chronic kidney disease

**DOI:** 10.1007/s10157-026-02818-9

**Published:** 2026-01-24

**Authors:** Raku Son, Takuya Fujimaru, Tatsuo Kagimura, Tadashi Sofue, Takao Masaki, Masaaki Nakayama, Ichiei Narita

**Affiliations:** 1 Department of Nephrology, St. Luke’s International Hospital, 9-1 Akashi-cho, Chuo-ku, Tokyo, 104-8560 Japan; 2 Foundation for Biomedical Research and Innovation at Kobe, Kobe, Japan; 3 Department of Cardiorenal and Cerebrovascular Medicine, Kagawa University, Kagawa, Japan; 4 Department of Nephrology, Hiroshima University Hospital, Hiroshima, Japan; 5 Division of Clinical Nephrology and Rheumatology, Kidney Research Center, Niigata University Graduate School of Medical and Dental Sciences, Niigata, Japan

**Keywords:** Chronic kidney disease (CKD), Erythropoietin, Mean corpuscular volume

## Abstract

**Background:**

Mean corpuscular volume (MCV) is routinely measured in patients with chronic kidney disease (CKD) and anemia; however, its prognostic significance, particularly in the context of erythropoiesis-stimulating agent (ESA) therapy, remains unclear.

**Methods:**

We conducted a post hoc analysis of the BRIGHTEN study, a multicenter, prospective trial that enrolled 1219 ESA-naïve patients with non-dialysis-dependent CKD who initiated darbepoetin alfa. Patients were categorized based on changes in MCV from baseline to week 16 as either increased or decreased. The primary outcome was renal function decline, defined as the initiation of dialysis, kidney transplantation, ≥ 50% reduction in estimated glomerular filtration rate (eGFR), or an eGFR ≤ 6 mL/min/1.73 m^2^ within 96 weeks.

**Results:**

MCV decreased in 778 (63.8%) patients during the study period. Changes in MCV were not correlated with baseline MCV values or ESA responsiveness. Over a mean follow-up of 2.46 ± 0.78 years, renal function decline occurred in 304 (39.1%) and 140 (31.7%) patients in the decreased and increased MCV groups, respectively. After adjusting for age, sex, baseline eGFR, albumin, high-sensitivity CRP, proteinuria, ferritin, transferrin saturation and ESA responsiveness, increased MCV remained independently associated with a reduced risk of renal function decline (adjusted hazard ratio 0.67; 95% confidence interval 0.53–0.85; *p* < 0.001).

**Conclusion:**

In ESA-naïve patients with non-dialysis-dependent CKD, an increase in MCV following ESA treatment was associated with a significantly lower risk of renal function decline. Monitoring MCV dynamics may serve as a simple, adjunctive tool for risk stratification and individualized CKD management.

**Supplementary Information:**

The online version contains supplementary material available at 10.1007/s10157-026-02818-9.

## Introduction

Anemia is a common complication of chronic kidney disease (CKD) and an important prognostic factor [1]. Mean corpuscular volume (MCV), routinely used to assess anemia causes, has been linked to mortality and renal outcomes in prior studies [2–8]. However, MCV may change after erythropoiesis-stimulating agent (ESA) initiation [9], and the relationship between these changes and renal outcomes is unclear.

The BRIGHTEN study, a multicenter, prospective trial investigating ESA responsiveness in patients with non-dialysis-dependent CKD [10], monitored changes in a comprehensive panel of anemia-related markers over 2 years in a unique cohort comprising only ESA-naïve patients. In the present study, we performed post hoc analysis of the BRIGHTEN dataset to determine whether changes in MCV following treatment with darbepoetin alfa (DA) were associated with renal outcomes.

## Methods

### Study design

This was a subanalysis of the BRIGHTEN study, a multicenter, prospective observational study involving 168 Japanese facilities. The main study design and results were previously described [10]. The protocol was approved by Nagoya University (approval no: 2014-0027) and all participating facilities and was registered at ClinicalTrials.gov (NCT02136563) and UMIN-CTR (UMIN000013464). Briefly, the study enrolled patients with non-dialysis-dependent CKD aged ≥ 20 years with a hemoglobin level of < 11 g/dL and no history of ESA use within the previous 12 weeks. A total of 1724 patients were included in the main analysis. For the present study, 505 patients with missing or incomplete data, including 168 patients with lacking data on renal outcomes, 13 patients without information on baseline MCV, and 324 patients without information on 16-week MCV, were excluded.

### Patient characteristics

The patient characteristics were compared using Student’s *t*-test, Mann–Whitney *U*-test, or the chi-square test, as appropriate. Data were presented as means ± standard deviation or medians [interquartile range], as appropriate. Erythropoietin resistance index (ERI)−1B was calculated by dividing the dose of darbepoetin alfa at 12 weeks (μg) by the hemoglobin level at 12 weeks (g/dL). Reciprocal ERI-2A was calculated as the increase in hemoglobin level from baseline to 12 weeks (g/dL) per unit of the total darbepoetin alfa dose during the 12 weeks (μg), which was then multiplied by body weight (kg) [10].

### MCV trajectory analysis and patient classification

The patterns of MCV trajectory during the first 16 weeks after the initiation of DA administration were analyzed using latent class mixed modeling (LCMM) implemented in the R package “lcmm.” The 16-week time point was selected because it represented the first evaluation after the stabilization of DA dosing, per the original BRIGHTEN study protocol [10]. Model fit with one to nine classes was evaluated using the Bayesian information criterion and the posterior probability of class membership. Models with three and four latent classes were identified as the most appropriate. Based on the results of the LCMM analysis, a clinically meaningful change in MCV was defined independent of cohort characteristics and used as an explanatory variable.

### Outcome analysis

Cumulative event-free survival for renal dysfunction was estimated using the Kaplan–Meier method, and differences were evaluated using the log-rank test. Cox proportional hazards regression with relevant covariates was performed to estimate adjusted hazard ratios with 95% confidence intervals. Model development was structured in three hierarchical steps to elucidate potential confounding or mediating effects of various clinical factors. Model 0 was an unadjusted model that included only MCV changes. Model 1 was adjusted for basic demographic and background variables, including age, sex, and baseline eGFR. Model 2 included the covariates in Model 1, as well as additional variables related to anemia or CKD progression: serum albumin, high-sensitivity C-reactive protein (CRP), urine protein-creatinine ratio (UPCR), ferritin, transferrin saturation (TSAT), and reciprocal ERI-2A. Cardiovascular events were additionally evaluated as an exploratory outcome, as the number of events was limited for robust multivariable modeling. The same definition of cardiovascular event was used as in the original BRIGHTEN study [10]. A two-sided *p* value of < 0.05 was considered statistically significant. All analyses were performed using R 4.1.3.

## Results

### Patient characteristics

The current study included 1219 of the 1,724 patients enrolled in the BRIGHTEN study, after excluding those with missing data (Figure S1). The cohort characteristics at enrollment are shown in Table 1. The mean age was 70.1 years, with 56.9% male patients. The average baseline eGFR was 20.6 ± 9.6 mL/min/1.73 m^2^, and the mean hemoglobin level and MCV were 9.8 ± 0.9 g/dL and 93.1 ± 5.7 fl, respectively. The median ferritin level was 94.5 ng/mL, and 15.2% of the patients were receiving iron supplementation at enrollment. During the first 12 weeks after enrollment, the patients received a total DA amount of 154.6 μg, with an average DA dose of 54.2 μg at 12 weeks. Patients excluded due to missing data were significantly more likely to be male and tended to have significantly lower kidney function and hemoglobin levels compared with those included in this subanalysis (Table S1).

**Table 1:** Characteristics of patients with increasing and decreasing MCV

	All	Decreased MCV	Increased MCV	*p*-value
n (%)	1219 (100)	778 (63.8)	441 (36.2)	
Progression of renal dysfunction (%)	444 (36.4)	304 (39.1)	140 (31.7)	0.013
Male (%)	694 (56.9)	428 (55.0)	266 (60.3)	0.082
Age (years)	70.1 ± 11.8	70.4 ± 11.6	69.6 ± 12.1	0.254
BMI (kg/m^2^)	23.1 ± 4.0	23.0 ± 4.1	23.1 ± 3.8	0.672
Creatinine (mg/dL)	2.7 ± 1.2	2.8 ± 1.3	2.7 ± 1.2	0.373
eGFR (mL/min/1.73m^2^)	20.6 ± 9.6	20.3 ± 9.5	21.2 ± 9.6	0.106
CKD etiology (%)				0.754
Nephrosclerosis	286 (23.5)	184 (23.7)	102 (23.1)	
Diabetic nephropathy	335 (27.5)	209 (26.9)	126 (28.6)	
Chronic glomerulonephritis	283 (23.2)	177 (22.8)	106 (24.0)	
Others	315 (25.8)	208 (26.7)	107 (24.3)	
CKD stage (%)				0.387
G2	2 (0.2)	2 (0.3)	0 (0.0)	
G3a	28 (2.3)	15 (1.9)	13 (2.9)	
G3b	152 (12.5)	92 (11.8)	60 (13.6)	
G4	629 (51.6)	399 (51.3)	230 (52.2)	
G5	408 (33.5)	270 (34.7)	138 (31.3)	
Urinary protein-creatinine ratio (g/gCr)	2.13 ± 2.74	2.14 ± 2.66	2.11 ± 2.89	0.867
Smoking status				0.404
Current (%)	121 (9.9)	82 (10.5)	39 (8.8)	
Ex-smoker (%)	443 (36.3)	270 (34.7)	173 (39.2)	
Systolic blood pressure (mmHg)	133.8 ± 18.9	134.9 ± 18.9	131.9 ± 19.0	0.008
Diastolic blood pressure (mmHg)	71.4 ± 12.3	71.7 ± 12.2	70.8 ± 12.4	0.219
HbA1c (%)	6.1 ± 0.9	6.1 ± 0.9	6.3 ± 1.0	0.015
Dyslipidemia (%)	667 (54.7)	438 (56.3)	229 (51.9)	0.158
Atherosclerosis (%)	411 (33.7)	252 (32.4)	159 (36.1)	0.216
RAS inhibitors (%)	793 (65.1)	499 (64.1)	294 (66.7)	0.408
Hypoglycemic agent (%)	384 (31.5)	239 (30.7)	145 (32.9)	0.299
Total dose of DA during 12 weeks (μg)	154.6 ± 92.1	160.1 ± 92.3	144.9 ± 90.9	0.005
Dose of DA at 12 weeks (μg)	54.2 ± 36.7	54.9 ± 37.1	52.9 ± 36.1	0.383
Iron supplementation at baseline (%)	185 (15.2)	97 (12.5)	88 (20.0)	0.001
Iron supplementation at 12 weeks (%)	380 (31.2)	203 (26.1)	177 (40.1)	< 0.001
Folic acid at baseline (ng/mL)	10.9 ± 34.8	11.6 ± 40.8	9.7 ± 20.4	0.375
Folic acid at 12 weeks (ng/mL)	10.0 ± 25.7	10.3 ± 29.7	9.5 ± 16.3	0.611
Vitamin B12 at baseline (pg/mL)	415.3 ± 229.6	422.1 ± 233.6	403.3 ± 222.2	0.183
Vitamin B12 at 12 weeks (pg/mL)	412.8 ± 230.5	428.7 ± 250.1	384.6 ± 187.5	0.002
NT-proBNP (pg/mL)*	472 [231–1050]	480 [236–1030]	446 [218–1100]	0.574
Serum albumin (g/dL)	3.74 ± 0.50	3.75 ± 0.50	3.72 ± 0.50	0.312
High-sensitivity CRP (ng/mL)*	546 [204–1675]	517 [188–1595]	614 [235–1815]	0.062
MCV at baseline (fL)	93.1 ± 5.7	94.0 ± 5.5	91.4 ± 5.8	< 0.001
MCV at 12 weeks (fL)	92.9 ± 5.5	92.6 ± 5.4	93.5 ± 5.6	0.01
Hemoglobin at baseline (g/dL)	9.8 ± 0.9	9.8 ± 0.9	9.8 ± 0.8	0.444
Hemoglobin at 12 weeks (g/dL)	11.1 ± 1.1	11.1 ± 1.1	11.2 ± 1.1	0.292
Serum iron at baseline (μg/dL)	70.7 ± 26.5	70.2 ± 22.7	71.7 ± 32.2	0.334
Serum iron at 12 weeks (μg/dL)	79.2 ± 27.5	77.7 ± 26.2	81.8 ± 29.4	0.019
Ferritin at baseline (ng/mL)*	94.5 [45.2–173.8]	91.6 [46.3–162.0]	102.5 [42.7–184.2]	0.59
Ferritin at 12 weeks (ng/mL)*	70.7 [41.8–128.0]	65.0 [38.9–123.0]	77.7 [45.4–135.0]	0.002
Transferrin saturation at baseline (%)	26.7 ± 10.1	26.6 ± 8.9	27.1 ± 12.0	0.368
Transferrin saturation at 12 weeks (%)	29.4 ± 10.5	28.8 ± 10.0	30.5 ± 11.4	0.007
ERI-1B [10]	5.0 ± 3.5	5.1 ± 3.6	4.9 ± 3.4	0.273
Reciprocal ERI-2A [10]*	0.51 [0.26–0.83]	0.50 [0.25–0.78]	0.59 [0.29–0.90]	0.002

Values are shown in mean ± standard deviation or median [interquartile range]

Statistical analyses were conducted using t-tests or chi-square tests as appropriate, except for values marked with an asterisk (*), which were analyzed using the Mann–Whitney U test

*BMI* body mass index, *CKD* chronic kidney disease, *CRP* C-reactive protein, *DA* darbepoetin alfa, *eGFR* estimated glomerular filtration rate, *ERI* erythropoietin resistance index, *MCV* mean corpuscular volume, *NT-proBNP* N-terminal pro B-type natriuretic peptide, *RAS* renin-angiotensin system

### MCV trajectory analysis and patient classification

In the overall cohort, MCV peaked at 6 weeks, with a subsequent nadir observed at 16 weeks (Figure S2). LCMM analysis during the first 16 weeks after the initiation of DA administration revealed that the changes in MCV followed a linear pattern, with patients clustering into increasing, stable, or decreasing trajectories, regardless of whether a three-class or four-class model was used (Figure S3). For clinical relevance, we defined two groups based on the net change in MCV between baseline and 16 weeks: the decreased MCV group (including those with stable MCVs) and the increased MCV group.

### Comparison of patients with decreased and increased MCV

During the study period, MCV decreased in 778 (63.8%) patients. As shown in Table 1, in patients with decreased and increased MCVs, the mean MCVs were 94.0 ± 5.5 and 91.4 ± 5.8 fl at baseline and 92.6 ± 5.4 and 93.5 ± 5.6 fl at 12 weeks, respectively. The mean systolic blood pressure was significantly higher and the mean HbA1c was significantly lower in the decreased MCV group than in the increased MCV group (134.9 ± 18.9 vs. 131.9 ± 19.0 mmHg, *p* = 0.008 and 6.1% ± 0.9% vs. 6.3% ± 1.0%, *p* = 0.015, respectively). Other clinical features, such as CKD stage and etiology, UPCR, history of atherosclerosis and dyslipidemia, use of renin-angiotensin system inhibitors, and levels of N-terminal pro B-type natriuretic peptide, serum albumin, and high-sensitivity CRP, were comparable between groups.

Some anemia-related parameters and treatment features were significantly different between the two groups. The cumulative DA dose during the first 12 weeks was significantly higher in the decreased MCV group than in the increased MCV group (160.1 ± 92.3 vs. 144.9 ± 90.9 μg, *p* = 0.005), although the DA dose at 12 weeks was comparable between the two groups (54.9 ± 37.1 vs. 52.9 ± 36.1; *p* = 0.383). This suggested greater ESA hyporesponsiveness in the decreased MCV group, indicated by a lower reciprocal erythropoietin resistance index (ERI)−2A.^8^ Compared with the decreased MCV group, significantly more patients in the increased MCV group received iron supplementation at baseline and 12 weeks. The median ferritin level at 12 weeks was significantly higher in the increased MCV group than in the decreased MCV group (77.7 [45.4–135.0] vs. 65.0 [38.9–123.0] ng/mL, *p* = 0.002), although iron deficiency was persistent in both groups. Albeit remaining within the normal range in both groups, the mean vitamin B12 level was slightly higher in the decreased MCV group than in the increased MCV group. At 12 weeks, hemoglobin at around 11 g/dL and TSAT at approximately 30% did not show significant differences. The changes in MCV were not correlated with the MCV absolute values or ESA responsiveness (Figure S4).

### Renal outcomes

**Fig. 1:**
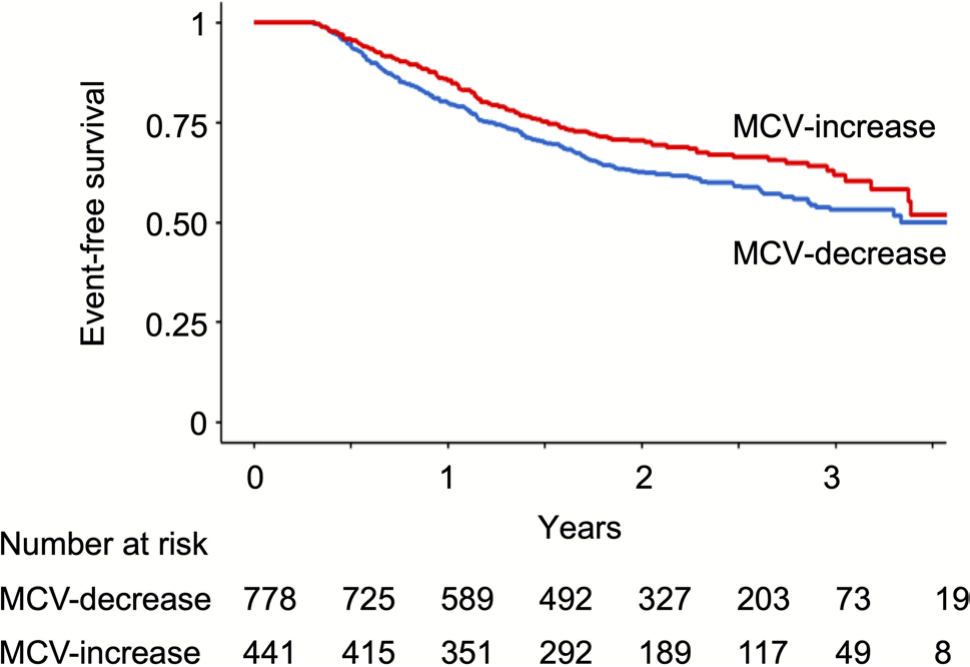
Kaplan–Meier analysis for renal prognosis in patients stratified by changes in MCV following darbepoetin alfa treatment

During a mean follow-up of 2.46 ± 0.78 years, 304 (39.1%) and 140 (31.7%) patients with decreased and increased MCV, respectively, experienced renal function decline, defined as the initiation of maintenance dialysis, kidney transplantation, 50% decrease in eGFR, or an eGFR ≤ 6 mL/min/1.73 m^2^. The event-free survival was significantly better in the increased MCV group than in the decreased MCV group (*p* = 0.02, log-rank test, Fig. 1). The risk for the progression of renal dysfunction was significantly lower in the increased MCV group than in the decreased MCV group, both in Model 1 (adjusted for basic demographic and background variables, including age, sex, and baseline eGFR; adjusted hazard ratio 0.76, 95% confidence interval [CI] 0.62–0.93, *p* = 0.008) and in Model 2 (further adjusted for variables related to anemia or CKD progression—serum albumin, high-sensitivity CRP, UPCR, ferritin, TSAT and reciprocal ERI-2A; adjusted hazard ratio 0.67, 95% CI 0.53–0.85, *p* < 0.001). In the component-specific analyses, none of the individual components of the composite kidney outcome was significantly associated with increased MCV (Table S2). In an exploratory analysis, changes in MCV were not significantly associated with cardiovascular events during follow-up (Table S3).

As a sensitivity analysis to further evaluate the clinical relevance of heterogeneous MCV trajectory patterns, we used the latent classes identified in the LCMM analysis—either three-class or four-class solutions—as explanatory variables to examine renal outcomes. Kaplan–Meier curves and multivariable Cox regression analyses were performed accordingly. In both the three- and four-class models, the class characterized by a decreasing MCV trajectory showed a consistently higher risk of renal function decline compared with classes exhibiting stable or increasing MCV patterns, supporting the robustness and clinical relevance of MCV trajectory–based risk stratification (Supplementary Figure S5 and Tables S4, S5).

## Discussion

We focused on changes in MCV following ESA treatment, unlike prior studies primarily focusing on baseline MCV [2–8]. This distinction is particularly relevant in CKD, where renal anemia is prevalent and ESA is frequently utilized. Given the confounding impact of ESA treatment on MCV, baseline MCV alone may not adequately reflect the erythropoietic dynamics, potentially explaining the inconsistencies in findings from earlier studies.

Our findings support the clinical utility of tracking the MCV trajectory. An increase in MCV may indicate more effective hematopoiesis with ESA treatment, driven by robust reticulocyte production [11]. Although the hemoglobin levels at baseline and 12 weeks did not significantly differ between the two groups, patients with decreased MCV might have had suboptimal iron availability or impaired iron utilization. Specifically, the frequency of iron supplementation, ferritin levels at 12 weeks, and TSAT were lower in patients with decreased MCV than in those with increased MCV, suggesting a blunted reticulocyte response associated with a decline in MCV despite ESA treatment. Monitoring MCV offers a simple, individualized marker of hematopoiesis, distinct from traditional indices, such as ERI. While ERI reflects hemoglobin changes, the MCV trajectory may be a more direct indicator of the quality of erythrocyte production and iron utilization, serving to determine whether timely adjustments to iron supplementation or other therapies in patients with decreasing MCV can aid in preserving renal function over the long term.

Several limitations merit consideration. First, evaluating the change in MCV at a single time point may not fully capture long-term variations. Second, although we adjusted for major confounders, the potential impact of residual factors, particularly reticulocyte indices, cannot be excluded. Future studies incorporating longitudinal reticulocyte measurements will be required to address this limitation. Third, all participants received DA, in accordance with the Japanese clinical guidelines, and the generalizability of our findings to other populations should be validated. Fourth, because patients with missing data were excluded from the analysis, the potential impact of selection bias cannot be ruled out.

In conclusion, evaluating MCV dynamics can help identify ESA-naïve patients with non-dialysis-dependent CKD who may be at higher risk for the progression of renal dysfunction, thereby improving clinical care through MCV-guided management strategies.

## Supplementary Information

Below is the link to the electronic supplementary material.

Supplementary file1 (PDF 1221 KB)

## Data Availability

The data that support the findings of this study are not publicly available due to privacy reasons but are available from the corresponding author upon reasonable request.
