# Supplementary material for: Changes in mean corpuscular volume after erythropoiesis-stimulating agent treatment are associated with renal outcomes in non-dialysis-dependent chronic kidney disease
Source: Clin Exp Nephrol. 2026 Jan 24;30(5):822–7. doi: 10.1007/s10157-026-02818-9 (PMC13090271; doi:10.1007/s10157-026-02818-9)

Supplementary Tables and Figures

Table S1. Characteristics of included and excluded patients.

	**Included**	**Excluded**	***p*-value**
n (%)	1219 (70.7)	505 (29.3)	
Progression of renal dysfunction (%)	444 (36.4)	239 (47.3)	<0.001
Male (%)	694 (56.9)	319 (63.2)	0.019
Age (years)	70.1 ± 11.8	69.5 ± 12.3	0.347
BMI (kg/m^2)	23.1 ± 4.0	23.4 ± 4.3	0.101
Creatinine (mg/dL)	2.7 ± 1.2	3.2 ± 1.6	<0.001
eGFR (mL/min/1.73m^2)	20.6 ± 9.6	19.1 ± 10.4	0.004
CKD etiology (%)			0.989
Nephrosclerosis	286 (23.5)	119 (23.6)	
Diabetic nephropathy	335 (27.5)	142 (28.1)	
Chronic glomerulonephritis	283 (23.2)	117 (23.2)	
Others	315 (25.8)	127 (25.1)	
CKD stage (%)			<0.001
G2	2 (0.2)	2 (0.4)	
G3a	28 (2.3)	16 (3.2)	
G3b	152 (12.5)	48 (9.5)	
G4	629 (51.6)	214 (42.4)	
G5	408 (33.5)	225 (44.6)	
Urinary protein-creatinine ratio (g/gCr)	2.1 ± 2.7	2.9 ± 3.8	<0.001
Smoking status			0.040
Current (%)	121 (9.9)	67 (13.3)	
Ex-smoker (%)	443 (36.3)	182 (36.0)	
Systolic blood pressure (mmHg)	133.8 ± 18.9	135.7 ± 19.6	0.084

Diastolic blood pressure (mmHg)	71.4 ± 12.3	71.2 ± 12.6	0.705
HbA1c (%)	6.1 ± 0.9	6.0 ± 0.8	0.096
Dyslipidemia (%)	667 (54.7)	276 (54.7)	1.000
Atherosclerosis (%)	411 (33.7)	166 (32.9)	0.778
RAS inhibitors (%)	793 (65.1)	322 (63.8)	0.649
Hypoglycemic agent (%)	384 (31.5)	166 (32.9)	0.109
Total dose of DA during 12 weeks (μg)	154.6 ± 92.1	144.4 ± 107.5	0.046
Dose of DA at 12 weeks (μg)	54.2 ± 36.7	58.5 ± 40.9	0.050
Iron supplementation at baseline (%)	185 (15.2)	65 (12.9)	0.065
Iron supplementation at 12 weeks (%)	380 (31.2)	120 (23.8)	0.002
Folic acid at baseline (ng/mL)	10.9 ± 34.8	11.6 ± 43.5	0.735
Folic acid at 12 weeks (ng/mL)	10.0 ± 25.7	12.5 ± 57.0	0.228
Vitamin B12 at baseline (pg/mL)	415.3 ± 229.6	414.3 ± 234.4	0.941
Vitamin B12 at 12 weeks (pg/mL)	412.8 ± 230.5	427.9 ± 248.7	0.269
NT-proBNP (pg/mL)*	472 [231–1050]	615 [280–1670]	<0.001
Serum albumin (g/dL)	3.7 ± 0.5	3.6 ± 0.6	0.001
High-sensitivity CRP (ng/mL)*	546 [204–1675]	674 [266–1950]	0.017
MCV at baseline (fL)	93.1 ± 5.7	92.9 ± 5.5	0.621
MCV at 12 weeks (fL)	92.9 ± 5.5	92.4 ± 5.6	0.078
Hemoglobin at baseline (g/dL)	9.8 ± 0.9	9.7 ± 1.0	0.024
Hemoglobin at 12 weeks (g/dL)	11.1 ± 1.1	10.7 ± 1.2	<0.001
Serum iron at baseline (μg/dL)	70.7 ± 26.5	70.0 ± 25.1	0.599
Serum iron at 12 weeks (μg/dL)	79.2 ± 27.5	76.6 ± 29.6	0.098
Ferritin at baseline (ng/mL)*	94.5 [45.2–173.8]	101.0 [49.7–185.8]	0.212
Ferritin at 12 weeks (ng/mL)*	70.7 [41.8–128.0]	81.0 [43.0–159.0]	0.008
Transferrin saturation at baseline (%)	26.8 ± 10.1	26.7 ± 9.5	0.828

Transferrin saturation at 12 weeks (%)	29.4 ± 10.5	29.1 ± 11.5	0.656
ERI-1B [10]	5.0 ± 3.5	5.7 ± 4.3	0.001
Reciprocal ERI-2A [10]*	0.5 [0.3–0.8]	0.4 [0.2–0.8]	0.010*

Values are shown in mean ± standard deviation or median [interquartile range].

Statistical analyses were conducted using t-tests or chi-square tests as appropriate, except for values marked with an asterisk (*), which were analyzed using the Mann–Whitney U test.

BMI, body mass index; CKD, chronic kidney disease; CRP, C-reactive protein; DA, darbepoetin alfa; eGFR, estimated glomerular filtration rate; ERI, erythropoietin resistance index; MCV, mean corpuscular volume; NT-proBNP, N-terminal pro B-type natriuretic peptide; RAS, renin-angiotensin system.

Table S2. Cox proportional hazards analysis showing hazard ratios associated with increased MCV for each component of the composite kidney outcome (Model 2).

Component	Hazard ratio	95% CI	*p*-value
Dialysis (n = 352)	0.83	0.65–1.08	0.164
Transplant (n = 15)	0.65	0.16–2.64	0.549
≥50% reduction in eGFR (n = 178)	0.77	0.54–1.10	0.152
eGFR ≤6 mL/min/1.73 m^2 (n = 135)	0.69	0.45–1.05	0.082

Patient with decreasing MCV was used as the reference group. The numbers of events in each component of the composite kidney outcome were shown (events may overlap across components). Hazard ratios were estimated using Cox proportional hazards models (Model 2), adjusted for basic demographic and background variables (age, sex, and baseline eGFR) as well as variables related to anemia or CKD progression (serum albumin, high-sensitivity C-reactive protein, urine protein-creatinine ratio, ferritin, transferrin saturation and reciprocal ERI-2A).

eGFR, estimated glomerular filtration rate; ERI, erythropoietin resistance index

Table S3. Cox proportional hazards analysis showing hazard ratios associated with increased MCV for cardiovascular events with increasing levels of covariate adjustment.

	Hazard ratio	95% CI	*p*-value
Model 0	1.38	0.94–2.03	0.1
Model 1	1.44	0.98–2.13	0.06
Model 2	1.19	0.75–1.89	0.47

Patient with decreasing MCV was used as the reference group.

Model 0, an unadjusted model that included only class MCV trajectories.

Model 1, adjusted for basic demographic and background variables, including age, sex, and baseline eGFR.

Model 2, included the covariates in Model 1, as well as additional variables related to anemia or CKD progression: serum albumin, high-sensitivity C-reactive protein, urine protein-creatinine ratio, ferritin, transferrin saturation, and reciprocal ERI-2A.

CI, confidence interval; CKD, chronic kidney disease; eGFR, estimated glomerular filtration rate; ERI, erythropoietin resistance index; MCV, mean corpuscular volume

Table S4. Cox proportional hazards analysis of the association between three-class MCV trajectory patterns and renal prognosis, with increasing levels of covariate adjustment.

	Hazard ratio	95% CI	*p*-value
Model 0			
Class 1 (n = 33)	1.00		
Class 2 (n = 1160)	2.26	1.07–4.78	0.03
Class 3 (n = 26)	3.58	1.41–9.11	0.007
Model 1			
Class 1 (n = 33)	1.00		
Class 2 (n = 1160)	1.66	0.78–3.51	0.19
Class 3 (n = 26)	3.37	1.32–8.62	0.001
Model 2			
Class 1 (n = 33)	1.00		
Class 2 (n = 1160)	1.53	0.68–3.47	0.31
Class 3 (n = 26)	3.66	1.33–10.13	0.01

Class 1 (increasing MCV trajectory) was used as the reference group in all models.

Model 0, an unadjusted model that included only class MCV trajectories.

Model 1, adjusted for basic demographic and background variables, including age, sex, and baseline eGFR.

Model 2, included the covariates in Model 1, as well as additional variables related to anemia or CKD progression: serum albumin, high-sensitivity C-reactive protein, urine protein-creatinine ratio, ferritin, transferrin saturation, and reciprocal ERI-2A.

CI, confidence interval; CKD, chronic kidney disease; eGFR, estimated glomerular filtration rate; ERI, erythropoietin resistance index; MCV, mean corpuscular volume

Table S5. Cox proportional hazards analysis of the association between four-class MCV trajectory patterns and renal prognosis, with increasing levels of covariate adjustment.

	Hazard ratio	95% CI	*p*-value
Model 0			
Class 1 (n = 39)	1.00		
Class 2 (n = 166)	1.62	0.76–3.43	0.21
Class 3 (n = 985)	2.33	1.16–4.70	0.02
Class 4 (n = 29)	2.87	1.17–7.02	0.02
Model 1			
Class 1 (n = 39)	1.00		
Class 2 (n = 166)	1.30	0.61–2.76	0.50
Class 3 (n = 985)	1.69	0.84–3.41	0.14
Class 4 (n = 29)	2.42	0.99–5.95	0.05
Model 2			
Class 1 (n = 39)	1.00		
Class 2 (n = 166)	1.20	0.53–2.74	0.66
Class 3 (n = 985)	1.47	0.69–3.14	0.32
Class 4 (n = 29)	2.24	0.86–5.88	0.10

Class 1 (increasing MCV trajectory) was used as the reference group in all models.

Model 0, an unadjusted model that included only class MCV trajectories.

Model 1, adjusted for basic demographic and background variables, including age, sex, and baseline eGFR.

Model 2, included the covariates in Model 1, as well as additional variables related to anemia or CKD progression: serum albumin, high-sensitivity C-reactive protein, urine protein-creatinine ratio, ferritin, transferrin saturation, and reciprocal ERI-2A.

CI, confidence interval; CKD, chronic kidney disease; eGFR, estimated glomerular filtration rate; ERI, erythropoietin resistance index; MCV, mean corpuscular volume

Figure S1. Participant flow diagram.

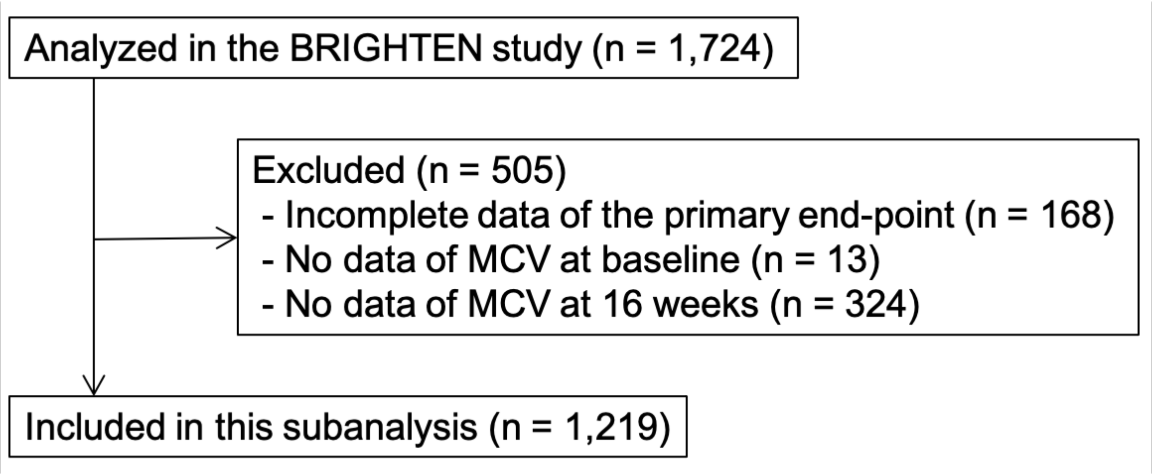

Figure S2. Trajectories of mean corpuscular volume (MCV) from enrollment to 96 weeks in the entire cohort.

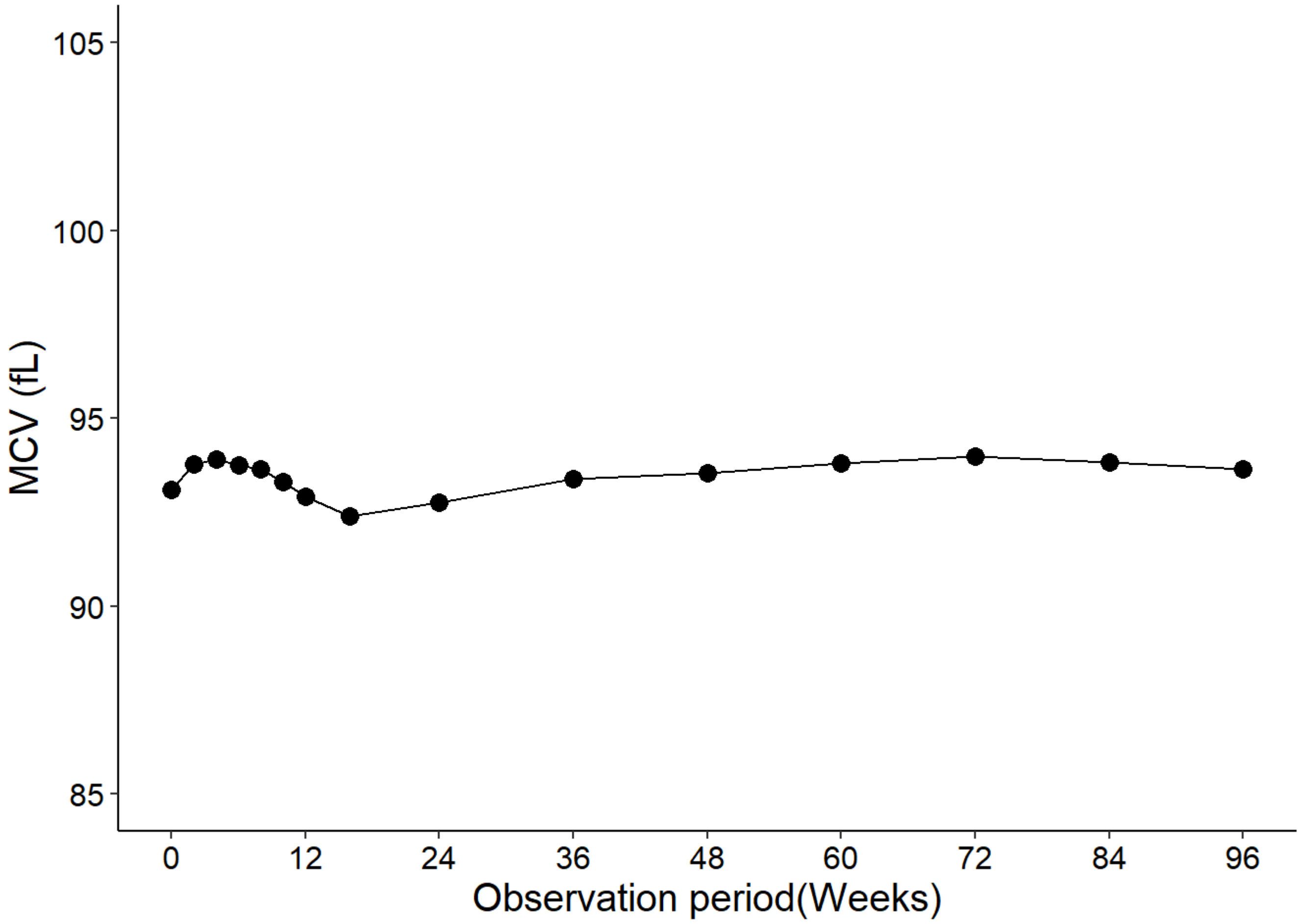

Figure S3. Patients categorized into (A) three and (B) four classes based on changes in MCV using latent class mixed modeling. Red lines represent class-specific mean predicted trajectories for each group.

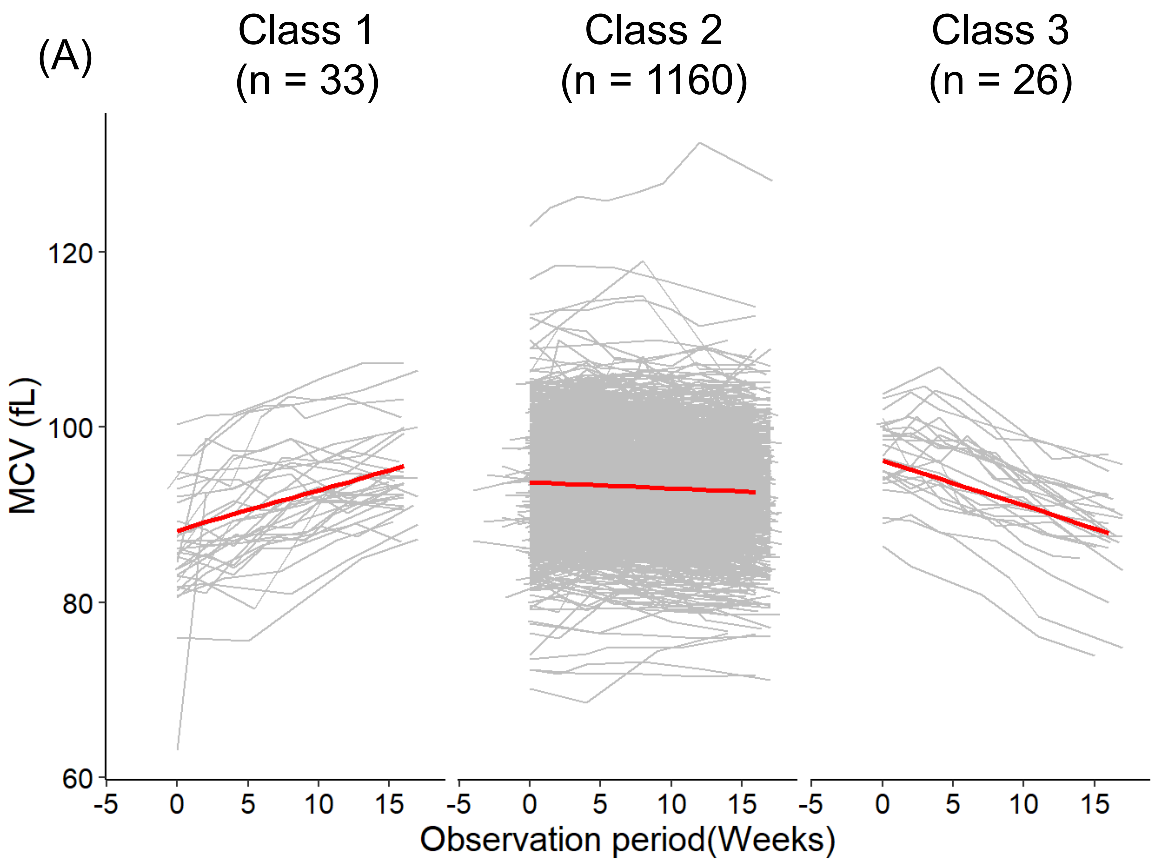

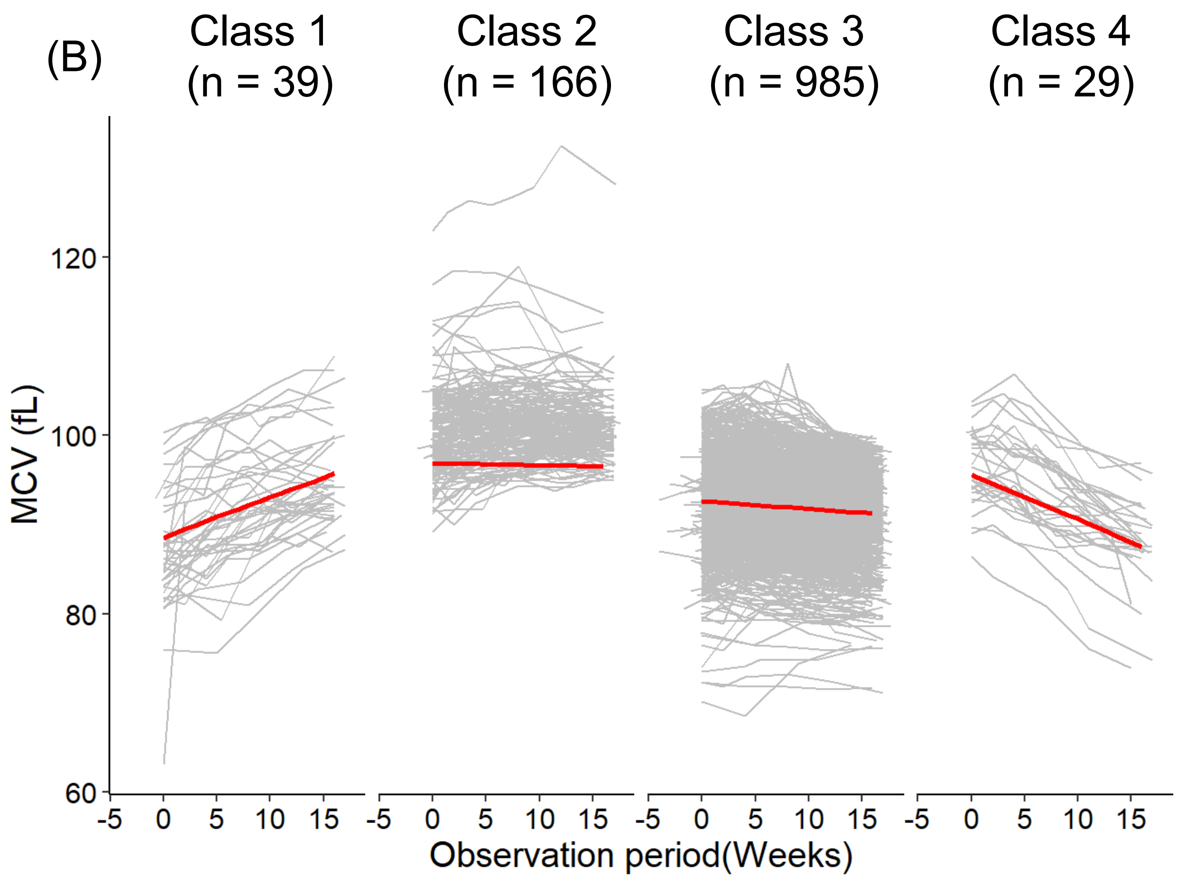

Figure S4. Correlation analysis examining the association of changes in MCV with the MCV absolute values (A) and reciprocal erythropoietin resistance index (ERI)-2A (B).

(A)

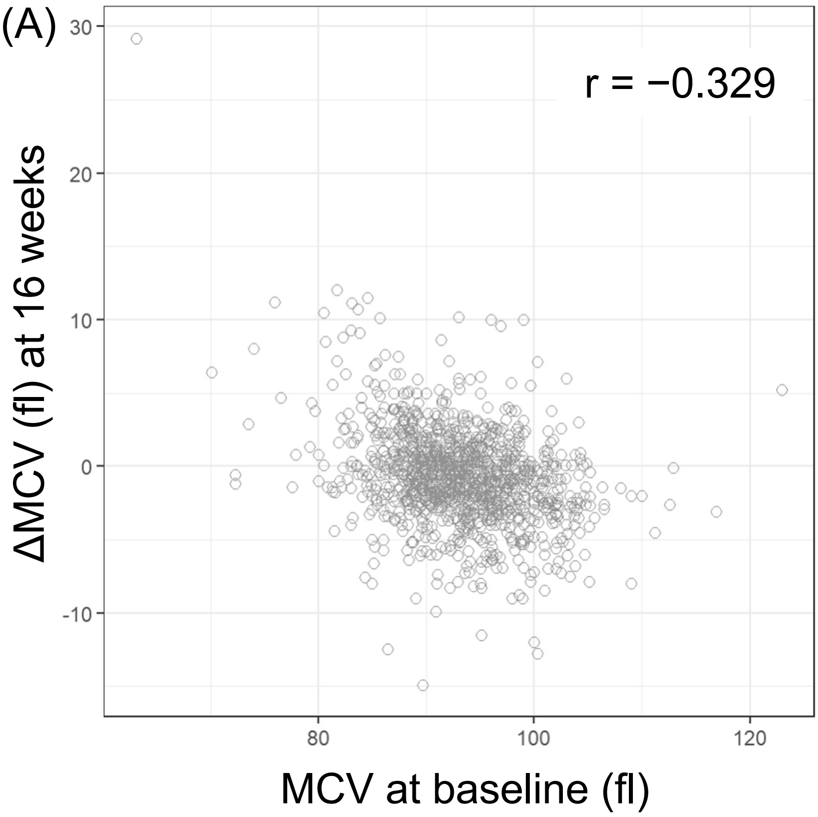

(B)

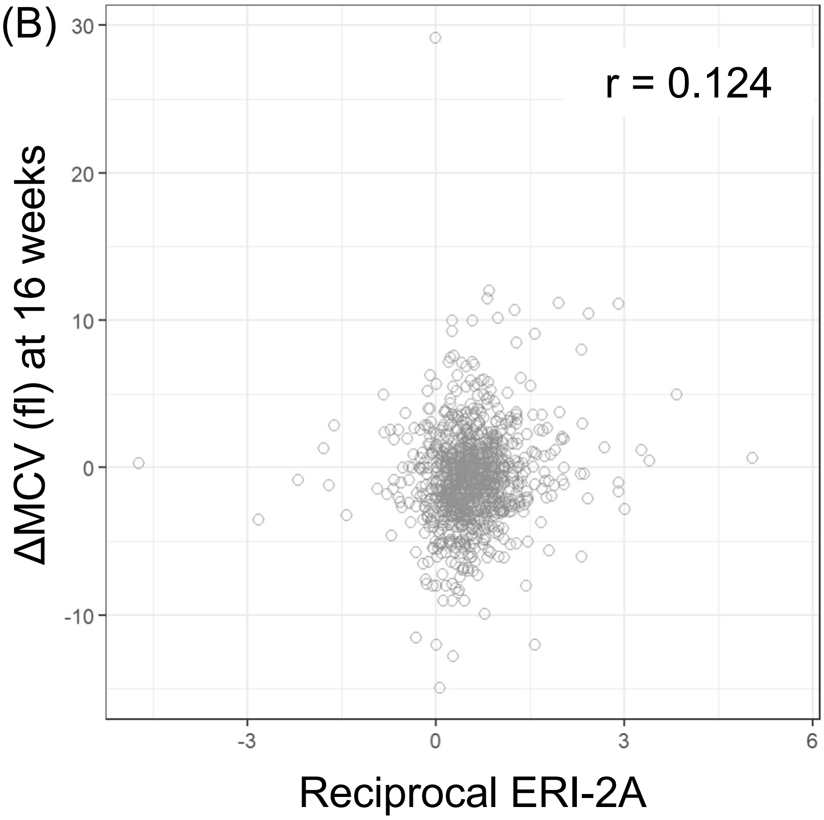

Figure S5. Kaplan–Meier analysis for renal prognosis in patients stratified by (A) three-class and (B) four-class MCV trajectories based on latent class mixed modeling.

(A)

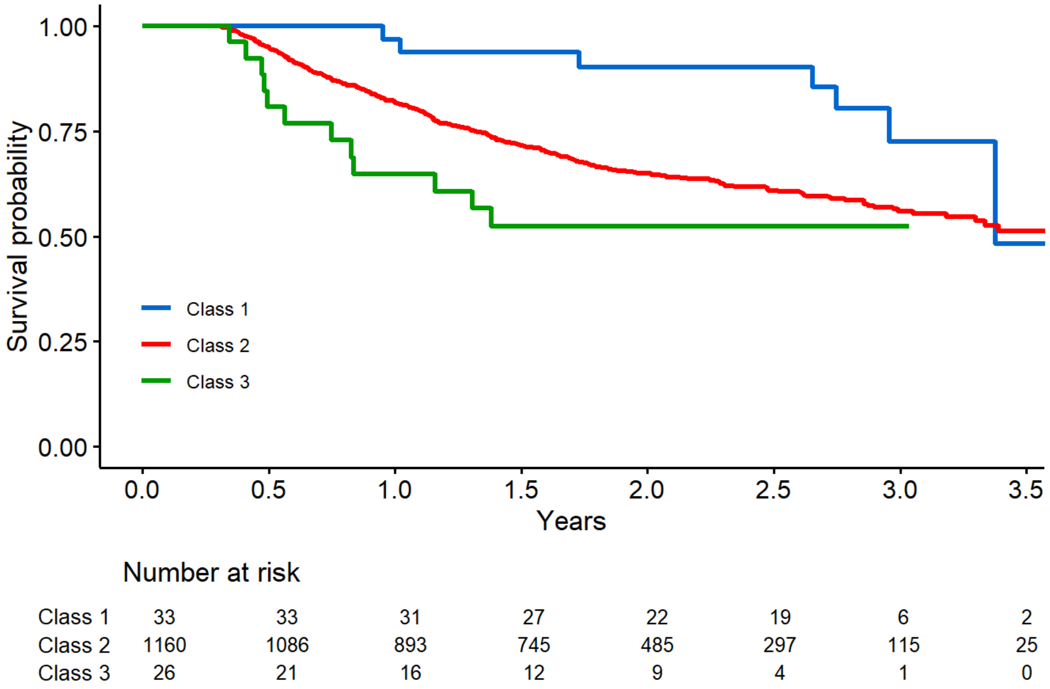

(B)

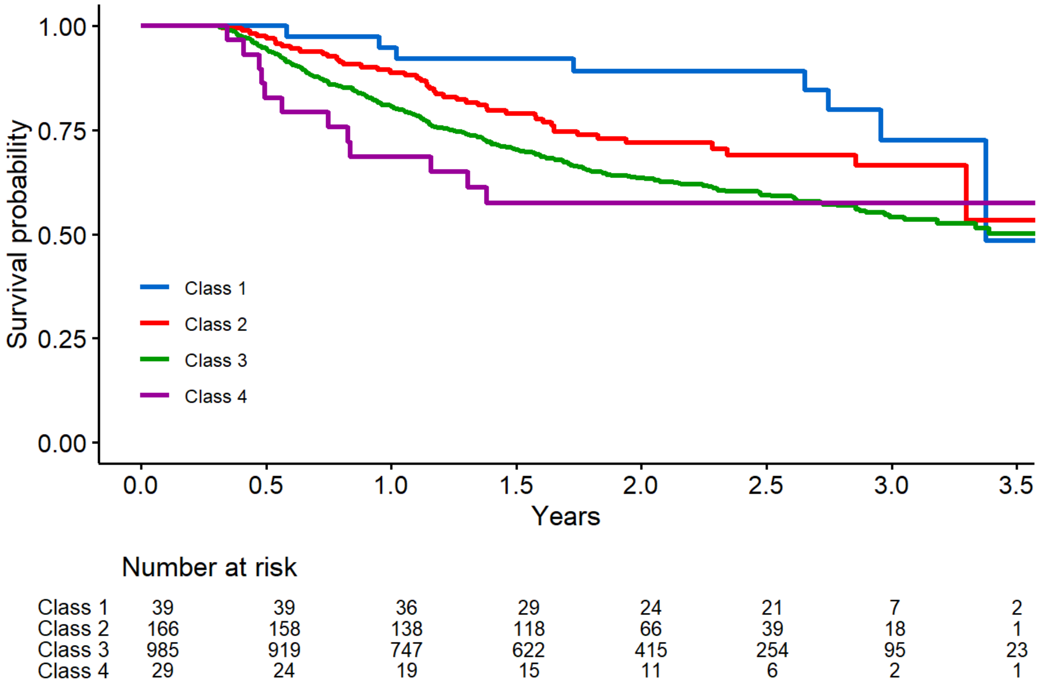

Supplement: Supplementary file 1 — Supplementary file1 (PDF 1221 KB) [file 10157_2026_2818_MOESM1_ESM.pdf]
